# Metformin Protects against Podocyte Injury in Diabetic Kidney Disease

**DOI:** 10.3390/ph13120452

**Published:** 2020-12-10

**Authors:** Sanna Lehtonen

**Affiliations:** Research Program for Clinical and Molecular Metabolism and Department of Pathology, Faculty of Medicine, University of Helsinki, 00290 Helsinki, Finland; sanna.h.lehtonen@helsinki.fi

**Keywords:** diabetes, diabetic kidney disease, glomerular epithelial cell, kidney, metformin, podocyte

## Abstract

Metformin is the most commonly prescribed drug for treating type 2 diabetes mellitus (T2D). Its mechanisms of action have been under extensive investigation, revealing that it has multiple cellular targets, either direct or indirect ones, via which it regulates numerous cellular pathways. Diabetic kidney disease (DKD), the serious complication of T2D, develops in up to 50% of the individuals with T2D. Various mechanisms contribute to the development of DKD, including hyperglycaemia, dyslipidemia, oxidative stress, chronic low-grade inflammation, altered autophagic activity and insulin resistance, among others. Metformin has been shown to affect these pathways, and thus, it could slow down or prevent the progression of DKD. Despite several animal studies demonstrating the renoprotective effects of metformin, there is no concrete evidence in clinical settings. This review summarizes the renoprotective effects of metformin in experimental settings. Special emphasis is on the effects of metformin on podocytes, the glomerular epithelial cells that are central in maintaining the glomerular ultrafiltration function.

## 1. Introduction

Metformin is the first-line medication for treating type 2 diabetes (T2D) [1]. It has been in clinical use for over 60 years, demonstrating its efficacy and safety [2]. As a monotherapy, it efficiently lowers blood glucose in a dose-dependent manner [3]. Metformin is also potent in combination with other therapies, a common practice in managing T2D, and typically the combination therapy leads to a greater reduction in hyperglycaemia than the use of each medication alone [4]. A common adverse effect of metformin therapy is gastrointestinal intolerance [5]. This can be relieved by either slowly increasing the administration dose, reducing the dose, or using slow-release preparations [1]. In addition, metformin may cause vitamin B12 deficiency in long-term use [6]. In rare cases, metformin causes lactic acidosis, the accumulation of lactic acid in the plasma [7], discussed in detail in the paragraph “7.3. Use of metformin when kidney function is impaired”. The great benefit of metformin is the lack of hypoglycaemia as an adverse effect, and it is also weight neutral and low-cost [2].

There are numerous studies and reviews on metformin covering the different actions of this multivalent drug, including the kidney. Here, I will take a cell biological, nephrocentric view on the actions of metformin. I will concentrate on its beneficial effects on podocytes, the glomerular visceral epithelial cells, in diabetic kidney disease (DKD). First, I will provide an overview of the nephron, the functional unit of the kidney, with special emphasis on podocytes. Next, I will describe DKD, including mechanisms that lead to its development, followed by an overview of the effects of metformin on podocytes at the cellular and molecular level in both podocytes in culture and in animal models of diabetes. Finally, I will highlight the effects of metformin as monotherapy and in combination therapy in T2D and DKD and discuss some of the open questions on the actions of metformin in podocytes.

## 2. Structure of the Nephron

**Figure 1:**
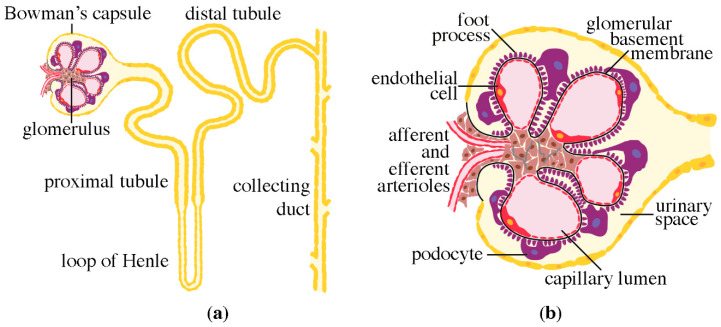
Schematic presentations of the nephron and the glomerulus. (**a**) Nephron, the functional unit of the kidney, consists of the glomerulus and the tubular compartment, which drains into the collecting duct system. (**b**) Glomerulus is the filtration unit of the nephron. Primary urine is filtered from the capillary lumen into the urinary space through the filtration barrier, consisting of the fenestrated endothelial cells, the glomerular basement membrane and the podocytes.

The nephron is the functional unit of the kidney [8]. Each nephron consists of a glomerulus, which is a tuft of capillaries, surrounded by a Bowman’s capsule, and a long, segmented tubule (Figure 1a). The tubular segments include the proximal convoluted tubule, the loop of Henle and the distal convoluted tubule, which drains into the collecting duct system (Figure 1a). The glomerulus has three distinct components: the fenestrated endothelial cells, the glomerular basement membrane, and the visceral epithelial cells or podocytes. These three components form together the glomerular filtration barrier [8] (Figure 1b). The function of this barrier is to ultrafiltrate plasma in a size and charge selective manner, allowing water, ions and proteins that are smaller in size than albumin (65 kDa) to pass, and retaining albumin and larger proteins in the circulation. In human, each kidney produces appr. 180 L of primary urine per day, which is then further modified in the tubular system via reabsorption of water, ions, and other components, to produce the concentrated urine to be excreted.

## 3. Podocytes, the Central Components of the Glomerular Filtration Barrier

**Figure 2:**
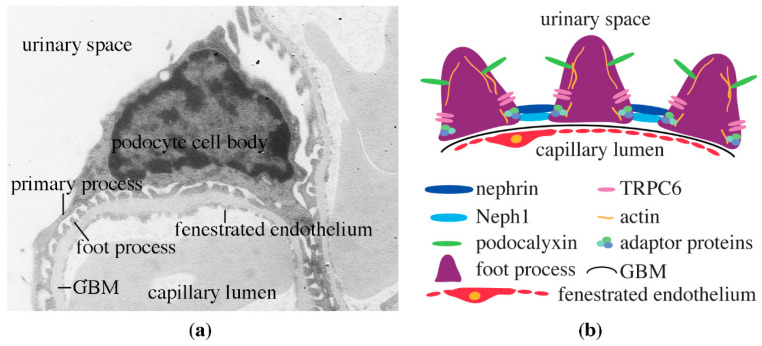
Micrograph of a podocyte and schematic presentation of the glomerular filtration barrier. (**a**) Electron micrograph of a podocyte, showing the cell body, primary process and foot processes. GBM, glomerular basement membrane. (**b**) The glomerular filtration barrier with its three layers: the podocytes, GBM and fenestrated endothelial cells. The cartoon visualizes two central slit diaphragm proteins, nephrin and Neph1 that associate with the actin cytoskeleton via adaptor proteins. Also TRPC6 and podocalyxin are depicted.

Podocytes are central in the maintenance of the kidney ultrafiltration function. They are terminally differentiated and thus, if lost, the function of the glomerular ultrafiltration barrier may get disturbed, leading to albuminuria. Podocytes are highly specialized cells with a peculiar morphology. They have a large cell body and primary processes that divide further into secondary and tertiary processes; the latter are also called foot processes (Figure 2a). The basal domains of the foot processes attach the podocytes to the glomerular basement membrane and interdigitate to efficiently cover the capillary loops (Figure 2a,b). Maintenance of the structural and functional characteristics of this podocyte domain is essential. As such, mutations in specific proteins in this domain or the underlying glomerular basement membrane could lead to nephrotic syndrome [9].

**Figure 3:**
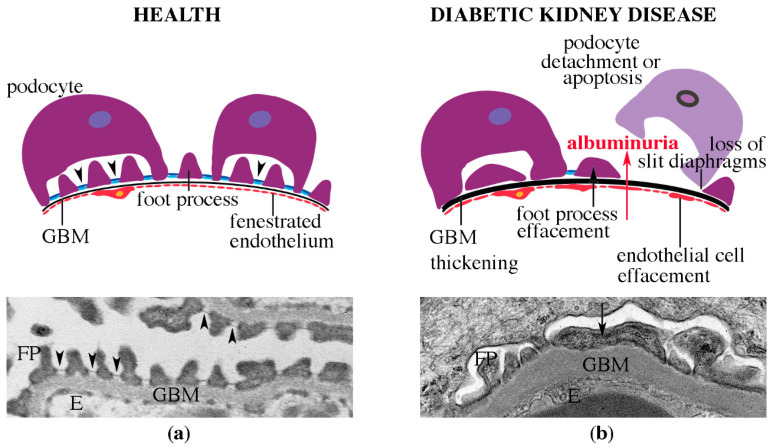
Morphological changes in the glomerular filtration barrier in DKD. (**a**) Schematic presentation (upper panel) and an electron micrograph (lower panel) of the glomerular filtration barrier in health. The three filtration barrier components, podocytes with their foot processes (FP), glomerular basement membrane (GBM) and endothelial cells (E) are well organized. The foot processes are interconnected by slit diaphragms (arrowheads). (**b**) Schematic presentation (upper panel) and an electron micrograph (lower panel) of the glomerular filtration barrier in DKD. GBM is thickened, podocyte foot processes (arrow) and endothelial cells are effaced, slit diaphragms are lost and podocytes are lost by detachment or apoptosis. DKD is characterized by albuminuria.

The foot processes of the neighboring podocytes form a specialized cell adhesion structure called the slit diaphragm (Figure 2b and Figure 3a). The slit diaphragm is an essential structural part of the glomerular filtration barrier, and in addition, it also plays a role as a signaling center (reviewed in [10]). The slit diaphragm is composed of a specific set of integral membrane proteins belonging to the immunoglobulin and cadherin superfamilies [10]. The central component is the immunoglobulin (Ig) superfamily member nephrin [11,12]. Nephrin was originally identified as a protein product of the gene *NPHS1*, which is mutated in the congenital nephrotic syndrome of the Finnish type [12]. Mutations in *NPSH1* lead to severe defects in kidney ultrafiltration and albuminuria [12]. In the slit diaphragm in vivo, a single molecule of nephrin spans the space between the podocyte foot processes creating a 45 nm wide diaphragm [11]. The lower, 23 nm wide part of the diaphragm, is formed by Neph1, another Ig domain-containing integral membrane protein. Together, nephrin and Neph1 create the flexible intercellular junction, the slit diaphragm [11] (Figure 2b and Figure 3a). Cryo-electron tomography of vitreous sections from mouse kidney shows very little trans-interaction between nephrin molecules in the adjacent podocyte foot processes [11]. This is further supported by studies using adhesion-deficient cells overexpressing nephrin [13]. Yet another component of the slit diaphragm protein complex is the canonical transient receptor potential 6 (TRPC6), which regulates Ca^2+^ dynamics in podocytes [14]. TRPC6 associates with nephrin and its interaction partner podocin, and interestingly, depletion of nephrin leads to an increase in the expression of TRPC6 in podocytes [14]. While activation of TRPC6 is essential in physiological conditions, an excessive increase in either TRPC6 expression or its channel activity may lead to pathological consequences via loss of podocytes in various forms of glomerular diseases, including DKD [15].

In addition to the transmembrane proteins, the slit diaphragm also contains a set of cytoplasmic adaptor proteins that associate with nephrin, mediate the signaling functions and connect the slit diaphragm protein complex to the underlying actin cytoskeleton (see, for example [16,17,18,19,20,21,22,23,24,25,26]; for review, see [10]). The actin cytoskeleton is central in maintaining the structural integrity of podocytes [27]. Nephrin is linked to the actin cytoskeleton via adapter protein CD2AP, which binds actin directly [28], and adaptor protein Nck, which associates with actin-regulating proteins [17]. The central role of actin in podocytes has been convincingly shown by mutating several actin-associated proteins, which lead to the disruption of the glomerular filtration barrier function and albuminuria [9,27].

Proteins at the apical domain of podocytes are equally essential for the normal glomerular function. Sialoprotein podocalyxin is an integral membrane protein with an extracellular anionic glycocalyx, providing a negative charge to podocalyxin [29]. The intracellular domain of podocalyxin associates with the actin cytoskeleton [29]. It has been proposed that the negative charge of podocalyxin keeps the filtration slits open by charge repulsion [30], and when disturbed, the structure of the podocytes is altered. In line with this, knocking out podocalyxin in mice specifically in podocytes during development leads to albuminuria and structural defects of the podocyte foot processes [31].

As described above, the elaborate structure and function of podocytes is maintained by a multitude of proteins at the apical and basolateral domains, at the slit diaphragm and in the intracellular compartment of podocytes. Mutations in several of these proteins, or alterations in their localization or expression level due to external factors, contribute to the disturbance in the kidney ultrafiltration function and lead to albuminuria. In line with this, changes in the expression of several of these proteins have also been observed in DKD [16,32,33,34]. Thus, maintenance of the normal localization and function of the podocyte proteins may help to hinder the development and progression of DKD.

## 4. Diabetic Kidney Disease

### 4.1. Clinical Definition of DKD

DKD is a potentially life-threatening complication of diabetes. It develops in approximately 30% of people with type 1 diabetes (T1D) and in 50% of people with T2D [35]. Clinical definition of DKD relies on the detection of albuminuria and estimated glomerular filtration rate (eGFR). DKD is diagnosed when either the urinary albumin/creatinine ratio exceeds 30 mg/g or the eGFR drops below 60 mL/min/1.73 m^2^, or when both criteria are fulfilled [35]. Even though albuminuria has been considered to be the hallmark of DKD, not all people with diabetes and renal dysfunction develop albuminuria. This concerns people with both T1D and T2D [35]. The progression of the disease may finally lead to the development of end-stage renal disease, requiring either dialysis or kidney transplant. In fact, DKD is the leading cause of end-stage renal disease, and it is associated with both increased risk of cardiovascular disease and mortality [35,36,37].

### 4.2. Pathomorphological Characteristics of DKD

An early morphological sign of DKD is the thickening of the glomerular basement membrane [38,39] (Figure 3). In addition, there is accumulation of mesangial matrix, which may develop to nodular glomerulosclerosis upon progression of the disease. Also tubulointerstitial fibrosis, tubular atrophy and arterial hyalinosis are observed [38,39]. Morphological alterations in podocytes include effacement, or flattening of the foot processes, and podocytes may be lost by apoptosis or detachment (Figure 3b) [39].

The morphological changes in the structure of the kidney are observed early on, before DKD is clinically characterized, indicating that renal biopsies would be highly beneficial in diagnosing DKD [40]. The structural changes in the kidney are associated with the clinical progression of the disease in both T1D and T2D [41,42], and interestingly, in people with T2D, the changes in albuminuria, but not in GFR, were found to associate with the early structural changes in the kidney [42].

### 4.3. Risk Factors of DKD

There are several both non-modifiable and modifiable risk factors that contribute to the development of DKD. The non-modifiable risk factors include, for example, increasing age, young age at onset of diabetes, long duration of diabetes and genetic factors [43]. The modifiable risk factors include, among others, poor glycaemic control, hypertension, dyslipidemia, smoking, obesity, sedentary lifestyle, low intensity of physical activity, insulin resistance or the metabolic syndrome [43]. In line with this, better management of diabetes has reduced the incidence of kidney dysfunction and development of end-stage renal disease [35].

### 4.4. Mechanisms Leading to the Development of DKD

**Figure 4:**
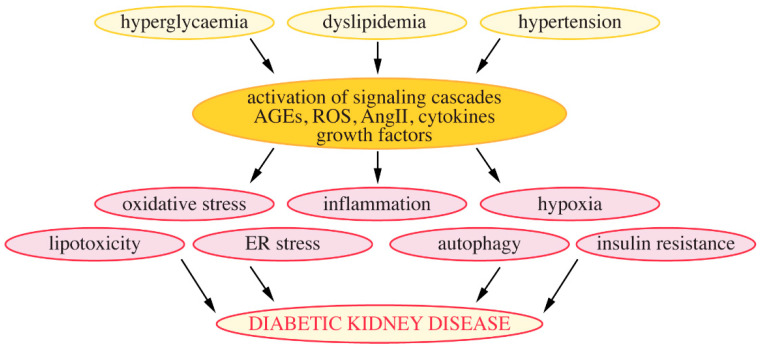
Mechanisms leading to the development of DKD. AGEs, advanced glycosylation end products; ROS, reactive oxygen species; AngII, angiotensin II; ER stress, endoplasmic reticulum stress.

The pathophysiology of DKD is complex. Hyperglycaemia, high blood pressure and dyslipidemia lead to the activation of various signaling cascades and production of cytokines and harmful substances, which affect various cellular processes that contribute to the development of DKD. These include, among others, oxidative stress, low-grade inflammation, hypoxia, lipotoxicity, endoplasmic reticulum stress, altered autophagic activity and insulin resistance (Figure 4). Podocytes as central components of the glomerular filtration barrier play a pivotal role in the pathogenesis of DKD [44]. Below, I will highlight cellular changes that can be targeted by metformin, concentrating specifically on those relevant in podocytes.

#### 4.4.1. Hyperglycaemia

A recent study reveals that in physiological conditions the central energy sources in podocytes are anaerobic glycolysis and fermentation of glucose to lactate [45]. Mitochondrial oxidative phosphorylation, with fatty acids used as key fuel, plays a minor role in podocytes [45]. In diabetes, hyperglycemia can lead to numerous glucotoxic effects in podocytes by activating various harmful pathways. One way how the podocytes can fight against the toxic effects of glucose is enhancement of the glycolytic flux, revealed in a study on individuals with long duration (>50 years) of diabetes who were protected from DKD. The study shows an increased expression and activity of the glycolytic enzyme pyruvate kinase M2 (PKM2) in the kidneys of these individuals [46]. PKM2 activation increases the metabolic flux of glucose, inhibits the production of glucose metabolites that are toxic to cells, and enhances the biogenesis of mitochondria [46].

High glucose can lead to non-enzymatic glycosylation of proteins leading to generation of advanced glycosylation end products (AGEs). The expression of receptor of AGEs (RAGE) is increased in podocytes of diabetic *db/db* mice concomitant with increased levels of vascular endothelial growth factor (VEGF), recruitment of inflammatory cells and increased glomerular permeability and albuminuria [47]. VEGF, produced by podocytes, can act on both podocytes and endothelial cells, and its different isoforms, such as the isoforms of VEGF-A, can have either protective or harmful effects on the glomerular function [48]. Activation of RAGE also contributes to the activation of mesangial cells, increased production of transforming growth factor β (TGF-β) and glomerular sclerosis [47]. TGF-β can also act on podocytes and induce their apoptosis [49]. These data indicate an important role for AGEs and activation of RAGE in the development of DKD [47], and also point out the importance of crosstalk between the three glomerular cell types, podocytes, endothelial cells and mesangial cells, in regulating the glomerular function.

Hyperglycaemia and the associated loss of podocytes is one of the key mechanisms leading to kidney injury in diabetes. High glucose has been shown to significantly increase the generation of reactive oxygen species (ROS) in podocytes in vitro, leading to the activation of the pro-apoptotic p38 mitogen-activated protein kinase and caspase-3, and podocyte apoptosis [50]. The synthesis of ROS was shown to occur via the plasma membrane NADPH oxidase and the mitochondrial pathway [50]. High glucose also activates the local renin-angiotensin system in podocytes leading to increased levels of angiotensin II, further adding to podocyte injury and apoptosis [51]. In line with this, the currently available treatments for DKD include angiotensin receptor blockers and angiotensin-converting enzyme inhibitors [44,52,53]. Furthermore, it has been shown that high glucose induces podocyte apoptosis in a TRPC6-dependent manner [54]. High glucose-induced ROS generation increases the channel activity of TRPC6, leading to an increase in the intracellular Ca^2+^ concentration and induction of apoptosis [54].

Podocytes are terminally differentiated cells and thus their loss is detrimental to kidney ultrafiltration. In animal models of T1D and T2D, the onset of hyperglycaemia coincides with an increase in podocyte loss, suggesting that podocyte loss is a central mechanism contributing to the progression of kidney injury in experimental models of diabetes [50]. Podocyte loss is relevant also in human DKD, as the podocyte number in people with T1D, independent of their age, is lower than in those who do not have diabetes [55]. Furthermore, in Pima Indians with T2D, depletion of podocytes predicts the progression of T2D [56].

#### 4.4.2. Lipotoxicity and Lipid Metabolism-Associated Regulation of Podocytes

Ectopic lipid accumulation is associated with lipotoxocity, and interestingly, in individuals with T2D and DKD, there is increased accumulation of intracellular lipids and lipid droplet formation in all glomerular cell types, including podocytes, as well as in tubular cells [57]. This is coupled with dysregulation of genes that regulate lipid metabolism, including up-regulation of genes involved in fatty acid uptake and down-regulation of genes of enzymes and transcription factors that control fatty acid oxidation or proteins that transport fatty acids to intracellular sites of oxidation. Also the expression of genes that hydrolyze circulating triglycerides are down-regulated and those that encode lipoprotein receptors are up-regulated. These changes lead to accumulation of triglycerides and neutral lipids in kidney cells. In addition, the expression of cholesterol efflux genes is decreased and cholesterol receptor genes increased, leading to accumulation of intracellular cholesterol [57]. The gene expression pattern of lipid-regulating genes correlates with GFR and inflammation, suggesting that altered lipid metabolism could contribute to the development of DKD [57].

A later study reveals that podocyte-specific depletion of ATP-binding cassette A1 (ABCA1), which regulates the efflux of cholesterol and phospholipids from cells, renders podocytes susceptible to DKD [58]. ABCA1 depletion leads to accumulation of cardiolipin, a mitochondrial phospholipid, and dysfunction of mitochondria. Activation of ABCA1 or inhibiting cardiolipin peroxidation, which is harmful for mitochondria, prevents podocyte injury and ameliorates DKD [58]. Another study reveals an interesting link between plasma membrane lipids and cell survival signaling in podocytes via regulation of the insulin receptor. Increased expression of lipid raft protein sphingomyelin phosphodiesterase acid-like 3b (SMPDL1b) in podocytes, as observed in diabetes, leads to reduced production of ceramide-1-phosphate, an active sphingolipid, and lowed expression of insulin receptor at the plasma membrane [59]. Administration of exogenous ceramide-1-phosphate restores insulin signaling in podocytes in vitro and ameliorates DKD in vivo [59]. A recent review covers the role of altered lipid metabolism highlighting the role of lipids in the development and progression of DKD [60].

#### 4.4.3. Chronic Low-Grade Inflammation

Inflammation is activated as an attempt to maintain cell and tissue homeostasis under stress or harmful conditions, but when prolonged, leads to tissue injury. In the diabetic milieu, oxidative stress, AGEs and angiotensin II contribute to the activation of inflammatory pathways. Circulating pro-inflammatory cytokines interleukin-1α (IL-1 α), interleukin-8 (IL-8) and interleukin-18 (IL-18) are increased prior to the development of albuminuria [61]. Also the urinary level of tumor necrosis factor α (TNF-α) is associated with the severity of microalbuminuria in individuals with T2D [62]. A study in mice reveals that inhibition of TNF-α decreases macrophage infiltration in the kidney and the levels of circulating inflammatory cytokines, and ameliorates histological changes typical of diabetic kidney injury [63]. TNF-α is also central in inducing the expression of monocyte chemoattractant protein 1 (MCP1) [63], and interestingly, high urinary MCP1 level can predict rapid loss in kidney function in individuals with T2D and DKD [64]. Furthermore, depleting TNF-α specifically from macrophages reduces albuminuria, ameliorates renal histological changes and reduces macrophage infiltration in the kidney [63]. Collectively, these data show that macrophage-derived TNF-α plays a central role in the development of DKD.

#### 4.4.4. Insulin Resistance

A seminal finding in the diabetes research field is the discovery of insulin resistance as a central factor increasing the risk of DKD [65]. This discovery by Ahlqvist et al. sprouts from classifying individuals with diabetes, based on six clinical variables, to five subgroups, each with distinct characteristics and risk of diabetic complications. The subgroup of individuals with severe insulin resistance is at a particularly high risk of DKD [65]. These individuals represent 10.6%–16.8% of people with diabetes, depending on the cohort analyzed [65].

At the cellular level, binding of insulin to its receptor activates an intracellular signaling cascade that leads to the phosphorylation of phosphatidylinositol 4,5-bisphosphate (PI(4,5)P2) to phosphatidylinositol 3,4,5-trisphosphate (PI(3,4,5)P3). This leads to phosphorylation (activation) of Akt and subsequently uptake of glucose into cells. Podocytes are also insulin sensitive [66] and can develop insulin resistance, as revealed by the inability of podocytes isolated from diabetic mice to respond to insulin stimulation by phosphorylating Akt [67]. In podocytes, the insulin signaling pathway is regulated by lipid phosphatases, namely phosphatase and tensin homolog deleted on chromosome 10 (PTEN) and SHIP2 [68,69]. Both act on PI(3,4,5)P3: PTEN hydrolyzes the 3′-phosphate and SHIP2 the 5′-phosphate, thus suppressing the activity of the insulin signaling cascade. Recent reviews cover the regulation of insulin signaling and development of insulin resistance in podocytes in more detail [70,71]. The role of insulin signaling in podocytes in maintaining the normal kidney function is highlighted by knocking out insulin receptor specifically from podocytes in mice [72]. These mice, despite being normoglycaemic, develop albuminuria and typical morphological signs of DKD indicating that intact insulin signaling is necessary for normal kidney function [72].

#### 4.4.5. Autophagy

Autophagy is a cellular process that degrades proteins and organelles via the lysosomal pathway, aiming to maintain cellular homeostasis. It is essential to overcome stages of starvation and to remove damaged and dysfunctional organelles. Podocytes have a high level of basal autophagy that maintains their normal cellular function [73]. Podocyte-specific ablation of autophagy-related 5 protein leads to age-dependent podocyte loss and glomerulosclerosis, and these mice have also increased susceptibility to induced glomerular injury [73]. In line with this, recent studies reveal that defective autophagy in podocytes escalates glomerular abnormalities in DKD [74,75]. High-fat diet fed mice with deficient autophagy specifically in podocytes develop massive albuminuria [75]. Furthermore, the tubulointerstitial damage is exacerbated in these mice, apparently due to the excessive increase in albuminuria [75]. These mice also present with foot process effacement and reduced number of podocytes [75]. Autophagy-deficient podocytes are more prone to undergo high glucose-induced apoptosis than control podocytes [74]. Also, streptozotocin-induced diabetic mice deficient in autophagy specifically in podocytes show more severe kidney dysfunction than the control diabetic mice. This includes more pronounced albuminuria, severely altered podocyte structure, exacerbated loss of podocytes and more severe ultrastructural alterations in the glomerular filtration barrier [74]. These data indicate that the autophagic activity of podocytes protects against DKD.

Two central regulators of autophagy in the kidney are the energy sensors AMP-activated protein kinase (AMPK) and sirtuin-1 (SIRT1). AMPK is the central regulator of cellular energy homeostasis that is activated when the cellular AMP/ATP level rises [76]. Activation of AMPK induces autophagy in podocytes by activating unc-51 like kinase 1 (ULK1) [77]. This occurs independently of the activation of mammalian target of rapamycin (MTOR), known to regulate podocyte hypertrophy, the early compensatory pathway after podocyte loss. These data suggest that activating either AMPK or ULK1 offers a therapeutic option to activate autophagy and prevent podocyte injury in DKD [77]. SIRT1 is a NAD-dependent deacetylase and its activity is necessary to induce autophagy in response to starvation [78]. Knockdown of Sirt1 in mice aggravates albuminuria and mitochondrial dysfunction in both diabetes- and adriamycin-induced podocyte injury, and this is due to reduced clearance of dysfunctional mitochondria by autophagy [79].

Interestingly, individuals with T2D and massive proteinuria exhibit reduced autophagic activity in podocytes compared to individuals with low levels of proteinuria, suggesting that defective autophagy contributes to disease progression [75]. These data suggest that activation of autophagy could be an efficient strategy to prevent the decline of kidney function in diabetes. A recent review provides an overview of the regulation of autophagy and the effects of antihyperglycaemic medications on the autophagic flux in the kidney in DKD [80].

## 5. Mechanisms Whereby Metformin Reduces Hyperglycaemia

Metformin has various mechanisms of action via which it reduces hyperglycaemia. It has become evident that metformin has multiple targets and that it acts via various pathways, and new mechanisms of action are described at an even pace. Metformin decreases hepatic glucose production mainly by reducing hepatic gluconeogenesis [81]. Metformin also acts as an insulin sensitizer. In the liver, metformin ameliorates lipid-induced insulin resistance by activating both AMPK and Akt [82]. In muscle and adipose tissue metformin enhances glucose uptake by activating the insulin signaling pathway and by regulating the translocation and activity of glucose transporters [83,84,85]. In the intestine metformin inhibits glucose absorption and increases glucose use, and also affects metabolism by altering the gut microbiome [86].

Metformin acts via both AMPK-dependent and -independent mechanisms. AMPK is activated when cells are deficient in energy, such as starved for glucose, and numerous studies show that dysregulation of AMPK associates with the metabolic syndrome, insulin resistance and T2D (reviewed in [87]). Either physiological or pharmacological activation of AMPK reduces glucose production in the liver, enhances glucose uptake and stimulates fatty acid oxidation, thus making it an ideal target to treat T2D [87]. An early study revealed that metformin reduces hepatic glucose production by activating AMPK, as an AMPK inhibitor abolished the effects of metformin [88]. However, the exact molecular mechanism via which metformin reduces gluconeogenesis remained elusive as this inhibitor was later on found to be nonselective for AMPK. In line with this, metformin has been shown to activate AMPK in an indirect manner: it inhibits complex I in the mitochondrial respiratory chain, leading to an increase in AMP/ATP ratio [89]. An AMPK-independent mechanism for metformin is supported by the finding that liver-specific deletion of AMPK α1/α2 or liver kinase B1, which phosphorylates Thr172 of the α-subunit of AMPK to activate AMPK, does not abolish the ability of metformin to reduce hyperglycaemia [90]. Metformin functions also by inhibiting the glucagon signaling pathway in the liver via cyclic AMP and protein kinase A, independently of AMPK [91]. In the liver, metformin binds directly and inhibits mitochondrial glycerophosphate dehydrogenase, the redox shuttle enzyme, leading to altered redox state and reduction in gluconeogenesis [92]. Another direct target of metformin is Src homology 2 domain-containing inositol 5′-phosphatase 2 (SHIP2) [85]. By inhibiting SHIP2, metformin enhances glucose uptake in muscle cells and protects podocytes from apoptosis. Interestingly, metformin does not inhibit SHIP2 in the liver [85]. As in other tissues, also in the kidney metformin acts via both AMPK-dependent and -independent pathways as described in the following paragraphs, in which I will concentrate on the effects of metformin in podocytes. For comprehensive reviews covering the effects of metformin in the kidney beyond podocytes, see the following recent review articles [93,94,95,96,97]. The mechanisms of action of metformin in different tissues are reviewed in [98].

## 6. Podocyte-Protective Mechanisms of Action of Metformin

### 6.1. Metformin Restores the Expression of Central Podocyte Proteins

The key podocyte protein nephrin has been shown to be either down-regulated or mislocalized from the plasma membrane to the cytoplasmic compartment in models of DKD [16,32,33]. Similarly, nephrin is either truncated or its trafficking to the plasma membrane is disturbed in patients with congenital nephrotic syndrome, both deleterious to the kidney function [99,100]. In a rat model of T2D, induced by low dose of streptozotocin coupled with high-fat diet, nephrin expression at both mRNA and protein level is decreased and its secretion into urine is increased [101]. Eight weeks treatment of these rats with metformin restored the morphological abnormalities, reduced albuminuria and nephrinuria, and restored nephrin mRNA and protein expression [101]. This is supported by a work using cultured podocytes in vitro, in which metformin normalized high glucose-induced down-regulation of nephrin [102].

The function of nephrin as both a structural and signaling molecule in the slit diaphragm (reviewed in [10,103]) provides various mechanistic insights in how restoring nephrin expression by administration of metformin could protect podocytes. Based on the central role of nephrin in the slit diaphragm [11,12], restoring diminished nephrin expression can be expected to provide stabilization of the structure of the slit diaphragm. In addition, nephrin plays a role in cell survival, as it associates with the p85 regulatory subunit of phosphoinositide 3-OH kinase (PI3K), and stimulates PI3K-dependent AKT signaling, essential for cell survival, in podocytes [104]. Thus, up-regulation of the expression of nephrin by metformin could enhance podocyte survival.

Also podocalyxin, expressed on the apical domain of podocytes, is down-regulated in podocytes in the streptozotocin and high-fat diet model of T2D at both mRNA and protein levels, and its excretion into urine increased [105]. Similar to the case of nephrin, metformin restores podocalyxin expression and reduces its excretion into urine [105]. Podocalyxin is down-regulated in streptozotocin-induced T1D rats, which also show down-regulation of the actin-binding protein ezrin and adaptor protein NHERF2 [34], which connect podocalyxin to the actin cytoskeleton. It is plausible that reduced level of podocalyxin in DKD has a fundamental effect on the organization of the actin cytoskeleton and restoring its expression back to normal helps to maintain the structural integrity of podocytes.

Excessive entry of Ca^2+^ into podocytes via TRPC6 may lead to podocyte injury and apoptosis in various forms of glomerular diseases, including DKD [15]. In cultured podocytes, metformin normalizes the high glucose-induced excessive expression of TRPC6 via AMPK activation [102]. This accords with a study showing that depletion of TRPC6 in streptozotocin-induced diabetic rats with Dahl Salt-sensitive background yields podocyte-protective effects [106].

### 6.2. Metformin Regulates the Dynamics of the Actin Cytoskeleton

A recent modeling study reveals that podocyte foot processes are very sensitive to changes in the dynamic organization of the actin cytoskeleton, which then reflects in altered podocyte foot process morphology and disturbed integrity of the filtration barrier [107]. Central actin regulators in podocytes include the small GTPases RhoA and Rac1, whose expression needs to be tightly controlled [107,108]. Treatment of cultured podocytes with high glucose reduces the activity of Rac1 and increases the activity of RhoA, and interestingly, treatment of the cells with metformin reverts this [102]. TRPC6 may be involved, as intracellular concentration of Ca^2+^ is important for maintaining the stability of the actin cytoskeleton [108]. Unbalanced activation of these small GTPases by modulation of the intracellular Ca^2+^ concentration may lead to structural changes in the cytoskeleton, and this can be prevented by lowering the excessive expression of TRPC6 by metformin [102].

p130Cas is an actin-associated scaffolding protein that is connected with proteins at the slit diaphragm and the basal domain of podocytes [109]. Angiotensin II-treatment of cultured podocytes reduces the expression level of p130Cas and leads to its perinuclear accumulation [109]. This is coupled with altered organization of the actin cytoskeleton. Metformin restores the localization and expression of p130Cas [109]. Further studies will be needed to define whether p130Cas is involved in podocyte damage in DKD.

### 6.3. Metformin Reduces Oxidative Stress

Metformin has been shown to reduce oxidative stress in the kidney in animal models of both T1D and T2D [110,111,112]. In streptozotocin-induced T1D diabetic rats, the level of the endogenous antioxidant, glutathione, is reduced and the production of ROS is increased [110]. Metformin restores both levels back to normal, coupled with restored expression of genes involved in oxidative defense, such as glutathione S-transferase alpha, oxidoreductase and catalase [110]. In a model of T2D, induced by high-fat diet and streptozotocin, metformin increases the renal level of endogenous antioxidant superoxide dismutase and decreases the level of malondialdehyde, a marker of oxidative stress [112]. All assays in both studies were carried out at the level of the whole kidney, leaving open the beneficial effects of metformin in podocytes. In spontaneously diabetic Torii rats, modeling non-obese T2D, metformin normalizes the level of the oxidative stress marker 8-OHdG in both urine and kidney tissue [111]. Notably, metformin reduces the level of 8-OHdG specifically in podocytes, associated with reduced podocyte loss by apoptosis [111]. An interesting finding is that metformin only slightly decreases blood glucose and HbA1c in these rats, indicating that protection of podocytes from apoptosis is independent of lowering hyperglycaemia [111]. In all three models, metformin ameliorates albuminuria [110,111,112].

Metformin reduces high glucose-induced ROS production in podocytes [113], and work on podocytes in culture has shed light on the molecular mechanisms. NAD(P)H oxidase is a central enzyme responsible for the generation of ROS in podocytes, and interestingly, metformin reduces the high glucose-induced excessive activity of NAD(P)H oxidase [113]. Metformin also activates AMPK in podocytes, proposing that it could be involved in the antioxidative pathway through which metformin acts [113]. A follow-up study by the same team revealed that metformin reduces the activity of ecto-ATPase, which leads to an increase in the extracellular concentration of ATP, which in turn, activates the purinergic P2 receptors [114]. Notably, when the activation of P2 receptors is prevented, also the effect of metformin on AMPK activation and NAD(P)H inhibition is abolished [114]. This suggests that metformin acts, at least partially, via purinergic signaling in podocytes by increasing extracellular ATP and activating the purinergic P2 receptors [114].

### 6.4. Metformin Ameliorates Dyslipidemia

In high-fat diet and streptozotocin-induced diabetes and DKD in rats, metformin lowers serum triglycerides, total cholesterol and low-density lipoprotein (LDL) cholesterol [112,115] and increases high-density lipoprotein (HDL) cholesterol [112]. Similarly, metformin lowers serum triglycerides and non-HDL cholesterol in individuals with newly diagnosed T2D [116], and LDL cholesterol in individuals with T1D [117]. In addition to reducing systemic hyperlipidemia, metformin appears to reduce tubular lipid droplet accumulation in a mouse kidney injury model induced by high-fat diet feeding, but this finding is based on visual evaluation only [118].

### 6.5. Metformin Reduces Inflammation

Both systemic and local renal inflammation may play a role in the development of DKD. CD74, a receptor for macrophage migration inhibitory factor (MIF) is expressed in podocytes and its expression is up-regulated in DKD [119]. MIF is also elevated in both the kidney tissue and urine in kidney diseases and activation of CD74 by MIF leads to an inflammatory response in podocytes [119]. A study on people with T2D compares urine levels of MIF and CD74 in individuals receiving either sulfonylurea or sulfonylurea and metformin for 24 weeks [120]. The study reveals that metformin add-on therapy reduces MIF-to-creatinine, CD74-to-creatinine and also podocalyxin-to-creatinine ratio in urine in addition to reducing albuminuria [120]. The authors conclude that metformin reduces the inflammatory signaling by MIF-CD74 in podocytes and could thus protect the podocytes from injury. Notably, there were no differences in the fasting blood glucose of HbA1c values between the groups indicating that this effect of metformin is independent of the glycaemic control [120].

Other studies have revealed anti-inflammatory effects of metformin in animal models. In a high-fat diet-induced model of obesity, metformin reduces the levels of pro-inflammatory markers TNF-α, IL-6, leptin, resistin, plasminogen activator inhibitor type-1 (PAI-1) and MCP1, and increases the level of anti-inflammatory adiponectin in serum [118]. This indicates that metformin ameliorates systemic inflammation. At the renal level, metformin reduces F4/80-positive macrophage infiltration [118], which could be due to the lowered level of MCP1. Metformin also reduces glomerular fibrosis as visualized by reduced expression of fibronectin and collagen I and IV [118]. In podocytes, metformin restores the diet-induced reduction of synaptopodin, an essential regulator of the actin cytoskeleton in podocytes, and ameliorates foot process effacement supporting podocyte-protective actions of metformin [118]. Alhaidet et al. determined the mRNA expression levels of inflammatory markers TNF-α and IL-6 in streptozotocin-induced diabetic rats, and found that metformin prevents the increase of both markers in the kidney, supporting an anti-inflammatory action for metformin [110].

### 6.6. Metformin Reduces Insulin Resistance

Systemic insulin resistance associates with a greater risk of DKD [65] and thus, reducing insulin resistance could be an effective strategy to prevent the development and progression of DKD. Interestingly, though, Ahlqvist et al. noticed that the use of metformin in individuals with T2D was low in the severely insulin resistant individuals even though they might have greatly benefited of metformin [65]. Several studies have shown that metformin ameliorates insulin resistance in various clinical settings (reviewed in [121]). In obese adolescents with hyperinsulinemia metformin improves metabolic control and reduces insulin resistance [122]. Another study on individuals with normal glucose tolerance who were hyperinsulinemic and had the metabolic syndrome reveals that metformin reduces insulin resistance and restores physiological insulin secretion [123]. This indicates the potential of metformin to ameliorate insulin resistance in individuals at high risk of T2D.

At the cellular level, metformin affects lipid phosphatases PTEN and SHIP2 that regulate insulin signaling in podocytes [68,69]. In primary rat podocytes, high glucose or knockdown of AMPK subunits increase the expression level of PTEN, and metformin reduces high glucose-induced PTEN expression [124]. The effect of metformin on PTEN is indirect as metformin does not reduce the catalytic activity of PTEN [85]. When cultured in high glucose, podocytes lacking PTEN maintain their ability to take up glucose in response to insulin stimulation, suggesting that the AMPK-PTEN signaling axis regulates insulin sensitivity of podocytes [124].

SHIP2 is up-regulated in adipose tissue, skeletal muscle and podocytes in rodent models of diabetes [68,125] and contributes to the development of insulin resistance. Metformin directly binds to and inhibits the catalytic activity of SHIP2 and thereby ameliorates insulin resistance [85]. Overexpression of SHIP2 in cultured human podocytes induces apoptosis [68] and metformin rescues podocytes from SHIP2 overexpression-induced apoptosis by restoring the lowered Akt activation [85]. Similar findings are obtained with metformin polymer (PolyMet) that is assembled with hyalurionic acid (HA) to PolyMet-HA nanocomplexes, designed with the aim to reduce the adverse effects and dosing frequency of metformin and to increase its bioavailability [126]. The commonly used human podocyte cell line AB8/13 is cultured continuously in high insulin [127], rendering them rather insensitive to insulin stimulation. Nevertheless, metformin increases basal glucose uptake in these cells, and metformin together with insulin increases glucose uptake even more that metformin alone [85]. Interestingly, the activity of SHIP2 is elevated in the kidneys of individuals with T2D receiving sulfonylurea or insulin compared to those who receive metformin or individuals without T2D [85]. The elevated activity of SHIP2 also associates with an increased level of podocyte loss, whereas individuals with T2D receiving metformin show less severe podocyte loss [85]. These data, combined with the data on cultured podocytes, suggest that metformin acts renoprotectively by reducing podocyte loss via activation of the insulin signaling cascade.

In primary rat podocytes cultured in high glucose, insulin-induced activation of AMPK and AKT is abolished, and also insulin-induced glucose uptake is reduced, indicating that high glucose induces insulin resistance in podocytes [124]. In rat primary podocytes under both normal and high glucose conditions, metformin increases basal glucose uptake, but metformin treatment together with insulin does not lead to a further increase in glucose uptake [124]. SIRT1 is also an important regulator of insulin sensitivity, as systemic knockout of Sirt1 leads to insulin resistance [128]. Furthermore, conditional deletion of Sirt1 from podocytes of the *db/db* mice aggravates DKD [128] supporting an essential regulatory role for SIRT1 in podocytes. Metformin restores high glucose-induced reduction of SIRT1 expression and activity in cultured primary rat podocytes [129]. AMPK and SIRT1 are known to regulate each other, and this is also observed in podocytes suggesting crosstalk between these pathways in the regulation of insulin sensitivity. It appears, though, that metformin activates AMPK and SIRT1 via different pathways [129]. Interestingly, under normal glucose conditions down-regulation of SIRT1 increases the permeability of podocyte monolayer to albumin, and this is prevented by metformin [129].

Another study reveals that high glucose induces mammalian target of rapamycin (mTOR) activation, in addition to reducing AMPK activation, in cultured human podocytes, and that metformin activates AMPK and inhibits mTOR [130]. mTOR forms two complexes, mTORC1 and mTORC2 that are biochemically and functionally different [131]. mTORC2 phosphorylates AKT, and AKT then indirectly activates mTORC1 signaling. Active mTORC1 negatively regulates insulin signaling by acting on insulin receptor substrate protein 1 (IRS-1) [131]. The study on human podocytes does not define specifically the activation of the different mTOR complexes, but shows that metformin treatment reduces high glucose-induced podocyte apoptosis and increases the expression of AKT [130].

### 6.7. Metformin Activates Autophagy

SIRT1 is a central regulator of autophagy, and in line with this, mouse embryonic fibroblasts deficient of Sirt1 fail to fully activate autophagy upon stimulation by starvation [78]. Increased expression of Sirt1, in turn, activates autophagy [78]. Metformin prevents down-regulation of SIRT1 expression in high-fat diet and streptozotocin-induced diabetic rat kidneys [132]. This is coupled with promotion of autophagy, reduction of the expression of the extracellular matrix proteins, reduction of oxidative stress and improvement of the morphology of the glomerular filtration barrier [132]. All these effects are blocked by the inhibition of SIRT1, indicating that metformin enhances autophagy and ameliorates DKD via SIRT1 [132]. Up-regulation of SIRT1 by metformin leads to up-regulation of forkhead box protein O1 (FoXO1), a substrate of SIRT1, which translocates to the nucleus and enhances transcription of genes involved in the enhancement of the autophagic flux [132]. Another study using high-fat diet and streptozotocin to induce diabetes and DKD in rats similarly found that metformin reduces oxidative stress and enhances autophagy in the kidney via AMPK/SIRT1-FoXO pathway [115]. However, neither of the studies above addressed whether metformin enhances autophagy specifically in podocytes but looked at autophagy in the kidney in general.

### 6.8. Effects of Metformin Outside of Podocytes

Metformin has beneficial effects outside of podocytes that also largely impact the function of the kidney and the progression of DKD. For example, metformin ameliorates structural glomerular lesions and reduces renal expression of TGF-β, known to contribute to mesangial expansion and tubulointerstitial fibrosis [112]. Metformin also affects oxygen homeostasis in the kidney. Chronic hypoxia, as observed in diabetes, is a central factor affecting tubulointerstitial fibrosis. Hypoxia-inducible factor-1α (HIF-1α) is known to promote hypoxia-induced renal fibrosis. In Zucker diabetic fatty rats, a model of T2D, metformin reduces renal hypoxia and the accumulation of HIF-1α [133]. A later study on streptozotocin-induced T1D diabetic rats shows that metformin reduces renal medullary hypoxia by inhibiting the uncoupling protein-2 [134]. In spontaneously hypertensive rats, metformin reduces proteinuria via activation of HIF-2α-VEGF-A signaling and up-regulation of VEGF-A, which plays an important role in the maintenance of the integrity of the glomerular filtration barrier by regulating the formation of the endothelial fenestrations [135]. Metformin also ameliorates the senescence of renal tubular cells in culture and in diabetic *db/db* mice by up-regulating miR-130a-3p and down-regulating STAT3, a target gene of miR-130a-3p involved in the pathogenesis of DKD [136]. For recent reviews on the beneficial effects of metformin in the kidney at large, the readers are referred to [93,94,95,96,97].

## 7. Metformin Monotherapy and Metformin-Based Combination Therapy in T2D and DKD

### 7.1. Comparison of Metformin and Other Antihyperglycaemic Medications in T2D and DKD

A meta-analysis including 204 studies (179 trials and 25 observational studies) compares the effectiveness and safety of the current antihyperglycaemic medications [4]. The comparison includes thiazolidinediones, metformin, sulfonylureas, dipeptidyl peptidase-4 (DPP-4) inhibitors, sodium-glucose cotransporter 2 (SGLT-2) inhibitors and glucagon-like peptide-1 (GLP-1) receptor agonists as monotherapy or metformin-based combination therapy [4]. The study reveals that metformin monotherapy is associated with lower long-term cardiovascular mortality in comparison with sulfonylurea monotherapy [4]. This finding is confirmed by a later retrospective cohort study on 174 882 individuals who are new users of metformin or sulfonylureas and who persist on their medication when their kidney function declines (eGFR < 60 mL/min/1.73 m^2^). The study reveals that use of metformin is associated with a lower risk of major adverse cardiovascular events [137]. The meta-analysis also reveales that HbA1c is reduced to a similar extent with the monotherapies with the exception of DPP-4 inhibitors, which are less effective than metformin in reducing HbA1c [4]. In addition, the two-drug combination therapies with metformin are more effective than metformin monotherapy in reducing HbA1c. Metformin, DPP-4 inhibitors, GLP-1 receptor agonists and SGLT-2 inhibitors reduce or maintain body weight while sulfonylureas, thiazolidinediones and insulin increase body weight. Most hypoglycemic events are associated with the use of sulfonylureas and most gastrointestinal adverse events with the use of metformin and GLP-1 receptor agonists [4]. In addition to intensive glucose control, the management of diabetes includes control of blood pressure and dyslipidemia as well as diet and life-style interventions [35], but details on these are beyond the scope of this review.

Several studies on animal models of diabetes and individuals with T2D and DKD have reported beneficial effects of metformin on albuminuria. Table 1 gives examples of studies on animal models reporting improvement of albuminuria by metformin. These include both genetic and induced models of diabetes and DKD in both rats and mice.

Studies reporting beneficial effects of metformin on albuminuria in humans are scarce as summarized in Table 2. However, some studies even report that metformin does not improve albuminuria. For example, Miyazaki et al. show in a 3-month double-blind, randomized, placebo-controlled study on individuals with T2D (29 participants) that metformin does not reduce albuminuria while thiazolidinedione rosiglitazone does [138]. Another five-year study on drug-naïve individuals with T2D (4351 participants) compares metformin, rosiglitazone and sulfonylurea glyburide [139]. The study reveals that albuminuria slowly rises with metformin during the five-year follow up, while with rosiglitazone and glyburide albuminuria declines during the first two years and then rises [139].

The rarity of studies showing reduction of albuminuria by metformin in human studies could be explained by the great heterogeneity in the nature of diabetes, and the realization that maybe the right treatment and the medical requirement of each person with diabetes, with specific pathophysiological features driving their disease, have not crossed paths. This is brought up in an excellent manner in the study by Ahlqvist et al., reporting that individuals who are severely insulin resistant and thus at high risk of DKD use less metformin than expected, even though they could greatly benefit from it [65]. Thus, the new classification of diabetes into subgroups with specific characteristics can be expected to help to provide the most beneficial medication to each individual, and to design randomized trials targeting specific subgroups of patients. These could include a study defining whether metformin provides renoprotection to the subgroup with individuals with severe insulin resistance and at high risk of DKD.

Several studies on metformin, comparing it with other antihyperglycaemic drugs, have been carried out to define its ability to ameliorate DKD or other diabetes-related outcomes. In overweight individuals with T2D (1704 participants), metformin reduces the risk of diabetes-related endpoints and is associated with fewer hypoglycemic periods and less weight gain than individuals receiving insulin or sulfonylureas [142]. A retrospective observational cohort study of individuals with T2D (10,426 participants) reveals that the use of metformin associates with lower rate of progression to end-stage renal disease and all-cause mortality in comparison to non-metformin users [143]. Another study on individuals with T2D (390 participants) does not observe a beneficial effect of metformin, as an add-on to insulin, on microvascular complications [144]. A five-year study on drug-naïve individuals with T2D (4351 participants) shows that eGFR first rises with metformin, rosiglitazone or glyburide treatments and then declines, remaining higher with rosiglitazone compared to the other two [139]. In all groups lowering of eGFR to under 60 mL/min/1.73 m^2^ is rare [139]. Of the antihyperglycaemic medications in use, SGLT2 inhibitors show the clearest renoprotective effects in clinical trials. In individuals with T2D at high risk of cardiovascular disease, SGLT2 inhibitor empagliflozin as an add-on to standard care is associated with slower progression of DKD and lower risk of progression to macroalbuminuria or clinically relevant renal outcomes compared to placebo [145]. Empagliflozin has also both short-term and long-term beneficial effects on albuminuria compared to placebo, irrespective of the status of albuminuria at baseline [146]. As an adverse effect, genital infections are reported more often in the empagliflozin group [145].

### 7.2. Use of Metformin When Kidney Function Is Impaired

Metformin is not metabolized but is cleared by the kidneys as such. In individuals with renal impairment, this may lead to a serious side effect of metformin, namely lactic acidosis [7]. This is rare, though, with less than 10 cases per 100,000 patient-years of exposure [7], but has raised concerns on the use of metformin and stimulated studies aiming to resolve the safety especially in individuals with kidney disease. The practice was to contraindicate metformin when the eGFR declined below 60 mL/min/1.73 m^2^. In 2016 the restrictions were released for those with mild to moderate kidney dysfunction with eGFR of 30–60 mL/min/1.73 m^2^ [147,148]. A study following the change in the guidelines found metformin safe and efficient when the dose was adjusted to the eGFR [149]. No conclusive evidence for an increase in lactic acidosis due to metformin was observed when analyzing the reported cases of lactic acidosis in the U.S. Food and Drug Administration´s Adverse Event reporting System, restricting the analysis to U.S. cases [150]. Support for this finding is provided by other studies [143,151,152]. Another research found; however that the use of metformin is associated with an increased risk of lactic acidosis in individuals with eGFR < 30 mL/min/1.73 m^2^ [153].

Earlier, metformin was found to be associated with a significantly higher risk of all-cause mortality in people with T2D and advanced chronic kidney disease (defined as eGFR < 30 mL/min/1.73 m^2^), and the risk was dose-dependent, compared to those who did not use metformin [152]. Later studies have revealed beneficial effects of metformin in individuals with less severe chronic kidney disease. When comparing people with diabetes and reduced kidney function (eGFR < 60 mL/min/1.73 m^2^) persisting on monotherapy, those who received metformin had a lower risk of major adverse cardiovascular events than those who received sulfonylurea [137]. In addition, in people with T2D and reduced kidney function (eGFR > 30 mL/min/1.73 m^2^), the use of metformin was associated with a lower risk of all-cause mortality and progression of end-stage renal disease [143]. However, in the case of acute kidney injury, the use of metformin should be omitted [154]. These data indicate that the much-feared lactic acidosis is a rare event but nevertheless, metformin users need continuous monitoring of the potential reduction of kidney function. The data also support the notion that adjusting the dose of metformin to the eGFR enables the use of metformin also when the kidney function is impaired, and that metformin should be contraindicated when eGFR falls < 30 mL/min/1.73 m^2^ [2]. For a more detailed description of clinical studies on the use of metformin in DKD the readers are referred to a recent review article [94].

## 8. Future Perspectives

### 8.1. Systemic Versus Podocyte-Specific Effects of Metformin on Dyslipidemia and Inflammation

In several cases the beneficial effects of metformin may stem from the effects at the systemic level, and the local effects on podocytes await clarifications. For example, metformin ameliorates dyslipidemia at the systemic level in both animal models [112,115] and individuals with T1D [117] or T2D [116]. However, cellular dyslipidemia could be an important contributor to DKD progression [60]. Indeed, both tubular and glomerular cells, including podocytes, accumulate lipids that increase the susceptibility of these cells to injury, e.g., apoptosis of podocytes [60]. One study reported that metformin reduces lipid droplet accumulation in kidney tubular cells [118]. It would be highly interesting to analyze whether similar phenomenon is observed in podocytes and whether such a direct action on podocytes would protect podocytes from injury.

There is an interesting regulatory link between the transcription factor nuclear factor kappa B (NF-κB), which is linked to the inflammatory response, and the slit diaphragm protein nephrin [155,156]. NF-κB up-regulates nephrin expression at the transcriptional level in human podocytes [156], and on the other hand, lack of nephrin activates NF-κB, and this activation mediates glomerular injury [155]. Introducing nephrin to cultured human podocytes, in turn, represses the activity of NF-κB [155]. As metformin increases the expression of nephrin, it would be interesting to define whether some of the anti-inflammatory effects of metformin in podocytes could be mediated via metformin-induced increase in the expression of nephrin and the consequent down-regulation of NF-κB, including the suppression of its target genes involved in the inflammatory response.

### 8.2. Mechanisms Whereby Metformin Reduces Insulin Resistance and Protects the Kidney and Podocytes

Studies in vitro and in Xenopus revealed that metformin can induce the tyrosine phosphorylation of insulin receptor to a similar level as insulin, but it just takes a longer time [157]. The work revealed that metformin inhibits protein tyrosine phosphatase 1B (PTP1B), which reduces the tyrosine phosphorylation of the insulin receptor to inhibit the actions of insulin [157]. PTP1B is considered a good target to treat insulin resistance, T2D and obesity [158], and thus, it would be interesting to determine whether metformin inhibits PTP1B also in podocytes. This could potentially provide a new mechanism whereby metformin ameliorates insulin resistance of podocytes.

A central finding is the ability of metformin to prevent podocyte loss. It is possible that metformin protects the kidney by reducing systemic insulin resistance, a risk factor for DKD [65], but the in vitro studies also propose direct podocyte-protective effects of metformin [85,130]. This is intriguing, as podocytes are terminally differentiated cells and therefore unable to proliferate, with their loss leading to kidney dysfunction. Proving the clinical kidney-protective action of metformin would, however, require a randomized controlled trial addressing this endpoint specifically.

### 8.3. Metformin and Senescence of Podocytes

As the kidney ages, the GFR and the number of nephrons decrease, and the renal cells acquire a senescent phenotype [159]. Podocytes and tubular epithelial cells of individuals with T2D and DKD show an accelerated senescent phenotype, indicating that diabetic milieu leads to hastened biological aging of these cell types [160]. Metformin ameliorates the senescence of renal tubular cells in culture and in diabetic *db/db* mice [136] raising the question whether metformin could also reduce senescence of podocytes. Another interesting question is whether this could occur by activating autophagy. Activation of autophagy has been shown to prevent senescence and promote longevity in various model organisms [161]. In T2D, however, the activation of autophagy is impaired in both kidney tubules and podocytes [80]. Metformin has been shown to activate autophagy at the level of the whole kidney [115,132], leaving open whether metformin could boost up autophagy in the podocytes and thus prolong their functionality and lifespan under the stressful diabetic conditions. This is a valid suggestion and worth studying further, as AMPK and SIRT1, the two proteins that are central in promoting autophagy in nutrient-deficient conditions [80], can be activated by metformin in podocytes [129,130].

## 9. Conclusions

**Figure 5:**
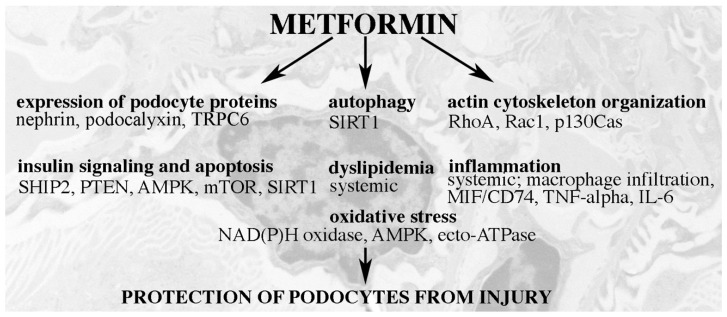
Cellular pathways via which metformin protects podocytes from injury in DKD.

Metformin beneficially affects various cellular pathways that are associated with the development of DKD (Figure 5). In line with this, in vitro work and numerous animal studies have shown that metformin protects both podocytes and kidney tubular cells from injury and hinders the development of DKD. However, whether metformin prevents clinical DKD is still an open question. As metformin has numerous indirect and also direct targets via which it modulates cellular functions, further studies are warranted to elucidate in more detail the exact mechanisms of action of metformin, including studies defining whether the effects on podocytes are cell-specific or mediated via improving systemic metabolism.

## Figures and Tables

**Table 1:** Examples of studies on animal models of diabetes and DKD showing reduction of albuminuria by metformin treatment.

Model	Metformin Administration	Reference
spontaneously diabetic Torii rat	350 mg/kg daily 17 weeks	[111]
high-fat diet and single low dose of streptozotocin rat	150, 300 or 500 mg/kg daily 8 weeks	[112]
high-fat diet and single low dose of streptozotocin rat	70 mg/kg daily 13 weeks	[101]
high-fat diet and single low dose of streptozotocin rat	250 mg/kg daily 8 weeks	[115]
high-fat diet and single low dose of streptozotocin rat	150, 300 or 500 mg/kg daily 8 weeks	[132]
streptozotocin rat	100 or 500 mg/kg daily 8 weeks	[110]
high-fat diet mouse	0.5% w/w in diet 12 weeks	[118]
*db*/*db* mouse	200 mg/kg daily 16 weeks	[136]

**Table 2:** Examples of clinical studies of diabetes and DKD showing reduction of albuminuria by metformin treatment.

Study/Cohort	Outcome	Reference
MARCH/newly diagnosed, drug-naïve individuals with T2D (*n* = 762)	Reduced albuminuria	[140]
Individuals with T2D with incipient nephropathy (switched from glybenclamide; *n* = 51)	Reduced albuminuria	[141]
Individuals with uncontrolled T2D (add-on to sulfonylurea; *n* = 202)	Reduced albuminuria	[120]
